# Rhizome Fragmentation by Vertical Disks Reduces *Elymus repens* Growth and Benefits Italian Ryegrass-White Clover Crops

**DOI:** 10.3389/fpls.2017.02243

**Published:** 2018-01-11

**Authors:** Björn Ringselle, Erik Bertholtz, Ewa Magnuski, Lars Olav Brandsæter, Kjell Mangerud, Göran Bergkvist

**Affiliations:** ^1^Department of Crop Production Ecology, Swedish University of Agricultural Sciences, Uppsala, Sweden; ^2^Division of Biotechnology and Plant Health, Norwegian Institute of Bioeconomy Research, Ås, Norway; ^3^Department of Plant Sciences, Faculty of Biosciences, Norwegian University of Life Sciences, Ås, Norway

**Keywords:** *Elytrigia repens*, *Trifolium repens*, *Lolium multiflorum*, mowing, cutting, mechanical weed control, perennial weed, intercrop

## Abstract

Tillage controls perennial weeds, such as *Elymus repens*, partly because it fragments their underground storage organs. However, tillage is difficult to combine with a growing crop, which limits its application. The aim of this study was to evaluate how soil vertical cutting with minimum soil disturbance and mowing affect the growth and competitive ability of *E. repens* in a grass–clover crop. A tractor-drawn prototype with vertical disks was used to fragment *E. repens* rhizomes with minimal soil and crop disturbance. In experiments performed in 2014 and 2015 at a field site close to Uppsala, Sweden, the rhizomes were fragmented before crop sowing (ERF), during crop growth (LRF), or both (ERF+LRF). Fragmentation was combined with repeated mowing (yes/no) and four companion crop treatments (none, Italian ryegrass, white clover, and grass/clover mixture). The results showed that in the grass–clover crop, rhizome fragmentation reduced *E. repens* rhizome biomass production and increased Italian ryegrass shoot biomass. ERF and LRF both reduced *E. repens* rhizome biomass by about 38% compared with the control, while ERF+LRF reduced it by 63%. Italian ryegrass shoot biomass was increased by 78% by ERF, 170% by LRF and 200% by ERF+LRF. Repeated mowing throughout the experiment reduced *E. repens* rhizome biomass by about 75%. Combining repeated mowing with rhizome fragmentation did not significantly increase the control effect compared to mowing alone. We concluded that rhizome fragmentation using vertical disks can be used both before sowing and during crop growth to enhance the controlling effect of grass–clover crops on *E. repens*.

## Introduction

A rotational grass or grass-legume crop that combines competitive perennial crops and periodic shoot cutting makes it difficult for annual weeds to establish and set seed and for perennial weeds to build up their storage organs (Teasdale et al., 2007). Shoot cutting retards plant development and biomass acquisition by severing valuable vegetative and propagative structures, and resets the light competition conditions. However, plants species are not equally sensitive to shoot cutting (Thomsen et al., 2015). For example, many grasses respond comparatively well to defoliation and can make up for lost biomass by compensatory growth (Ferraro and Oesterheld, 2002). By losing less than their neighbors from shoot cutting, plants can gain a competitive advantage by it. Many perennial weeds are sufficiently competitive and robust to shoot cutting, to propagate within grass-legume crops (Vanhala et al., 2006; Thomsen et al., 2015). Some weeds lower the quality and yield of grass-legume crops by replacing the more nutritious, more palatable, and higher-yielding forage crops. A few perennial weeds, e.g., couch grass [*Elymus repens* (L.) Gould], can also cause severe problems in subsequent crops, with associated control and environmental costs. Non-chemical methods are needed that selectively target these weeds without damaging the crop, combining injury to the weed with an increased competitive advantage for the crop.

Fragmentation of underground storage organs (e.g., rhizomes or thickened roots) is an important aspect of the mechanical control of perennial weed species. In perennial plants, clonal integration is generally beneficial to the clonal network as a whole, as ramets share resources (Song et al., 2013; Lopp and Sammul, 2016) and information (Stuefer et al., 2004). Fragmenting the storage organs severs these connections and can increase intraspecific competition, as reported by, Proctor (1972). However, many clonal species naturally fragment as part of their development (Hay and Kelly, 2008). Thus, fragmentation does not necessarily reduce biomass acquisition by itself, but divides the resources of the underground network, reducing the amount available to each fragment (Kolberg et al., 2017). This makes them more vulnerable to other effects of mechanical control, e.g., burial of fragments at greater depths (Håkansson, 1968). Moreover, while pathogens can spread through clonal networks (Stuefer et al., 2004), it is also likely that plant parts that are damaged are more susceptible to infections, e.g., by fungi (Imathiu et al., 2009), than intact plants.

Apart from cost and environmental impact, another major downside of tillage is that the destructive nature of most mechanical control methods makes them difficult to combine with a growing crop. However, a minimal-till approach that only fragments the storage organs of perennial weeds, without damaging the crop, could potentially enhance the competitive advantage of the crop over the weed. It may be difficult to reach the deep underground networks of perennial weeds such as Canadian thistle (*Cirsium arvense* L.) and coltsfoot (*Tussilago farfara* L.) with such an approach. However, for perennial weeds with stolons, shallow rhizomes, or shallow creeping roots [e.g., creeping bentgrass (*Agrostis stolonifera* L.), redtop (*Agrostis gigantea* Roth), *E. repens*, and field sow thistle (*Sonchus arvensis* L.)], it could provide farmers with an additional tool for weed control in growing crops, at a relatively low cost and environmental impact. For example, Bergkvist et al. (2017) found that when *E. repens* rhizomes were fragmented in a 10 cm × 10 cm grid using a flat spade in a newly established white clover sward, rhizome production was reduced by up to 60% compared with only white clover competition.

*Elymus repens* is a highly competitive perennial rhizomatous grass. It is a problematic weed in the temperate climatic zone (e.g., North Europe, North America, temperate Asia, Australia, and New Zealand), where it causes yield losses in a wide variety of annual and perennial crops. The two main control methods for *E. repens*, intensive tillage (Meyer et al., 1999; Aronsson et al., 2015) and herbicides (Heap, 2014; Bourguet and Guillemaud, 2016), both have serious potential negative environmental effects. Thus, if *E. repens* could be controlled in a rotational grass-legume crop without intensive tillage or herbicides, this would reduce the costs and environmental impact of the cropping system as a whole.

At sufficiently high crop biomass levels, grasses and legumes under-sown in cereals can significantly reduce *E. repens* rhizome biomass acquisition (Cussans, 1972; Bergkvist et al., 2010). However, the effect decreases with decreasing crop biomass (Ringselle et al., 2017). Thus, in sparse crop stands, the crops have little effect on *E. repens* rhizome biomass acquisition (Brandsæter et al., 2012; Melander et al., 2013; Ringselle et al., 2015). In such stands, legumes can even increase *E. repens* rhizome biomass in the following year, most likely due to their fertilizing effect (Ringselle et al., 2015). The extent to which crops suppress *E. repens* rhizome acquisition is influenced by their primary mode of resource competition. In pot trials, Ringselle et al. (2017) found that red clover (*Trifolium arvense* L.) reduced *E. repens* rhizome biomass more per gram biomass than perennial ryegrass (*Lolium perenne* L.), presumably because red clover competed more for light and ryegrass more for nutrients. Perennial ryegrass shifted *E. repens* biomass allocation toward belowground biomass, which signals competition for nutrients (Poorter et al., 2012). If clovers are particularly efficient at reducing the storage organs of perennial weeds, alone or in a mixture, this would provide an additional reason to use them as cover crops and in grassland mixtures. However, this has yet to be convincingly established in field trials.

Repeated shoot cutting can reduce *E. repens* rhizome biomass and carbohydrate acquisition, but it is relatively resilient to cutting compared with many other grasses (Harrison and Hodgson, 1939; Grassler and Von Borstel, 2005). A very high shoot cutting frequency, around every 2 weeks, is necessary to prevent *E. repens* from producing new rhizomes (Turner, 1966; Håkansson, 1969). Moreover, there appears to be seasonal variation in shoot cutting efficacy (Bergkvist et al., 2017). Consequently, most field experiments have found low and/or inconsistent effects of shoot cutting on *E. repens* rhizome biomass acquisition (Brandsæter et al., 2012; Ringselle et al., 2015). Cussans (1973) found that even cutting up to seven times per year in a perennial ryegrass sward did not fully prevent *E. repens* from propagating. Likewise, Lötjönen and Salonen (2016) found that shoot cutting seven times from July to October, in a timothy-meadow fescue ley, only provided moderate suppression of *E. repens*. Combining shoot cutting and competition has been shown to have a stronger effect than either factor on its own (Kolberg et al., 2017) but, depending on the crop, mowing can have an equally negative effect on the crop as on *E. repens* (Ringselle et al., 2015). This, combined with economic costs and time restraints, means that it is not feasible to apply a very high cutting frequency. Adding rhizome fragmentation could be one way to improve *E. repens* control without increasing the shoot cutting frequency.

The overall aim of this study was to develop a method for controlling *E. repens* without using herbicides and with a minimal-till approach that would be unlikely to cause nutrient leaching or damage to the crop. In a field experiment, *E. repens* was managed in a grass–clover sward with a combination of rhizome fragmentation and repeated mowing. The hypotheses tested were that: (1) rhizome fragmentation reduces *E. repens* and increases crop production in a grass–clover crop, (2) the effect on *E. repens* is enhanced by repeated mowing, and (3) competition from white clover reduces *E. repens* rhizome biomass relatively more, and shoot biomass relatively less, than Italian ryegrass. The rhizomes were fragmented using a recently developed prototype, the Kverneland vertical rhizome/root cutter (tractor-drawn), which uses vertical coulter disks taken from a plow to fragment the rhizomes with minimal soil disturbance.

## Materials and Methods

### Site

The experiment was performed in 2014 and 2015 using different parts of a field located south of Uppsala, Sweden (N 59°44′, E 17°39′). The field had an established *E. repens* population and is certified organic (by KRAV, a Swedish organic certification organization) and mechanical treatments are performed regularly, including tillage to control perennial weeds. To avoid interference from other perennial weeds, sections of the field with an even distribution of *E. repens* and as low a population of other perennial weeds as possible were selected. The annual weed species composition was not assessed. The previous crop was barley for the 2014 experiment and mown fallow for the 2015 experiment (the fallow was intended to preserve the *E. repens* population while controlling other perennial weeds). Autumn plowing was performed in 2013 and spring harrowing in 2014, while plowing and harrowing were both performed in spring 2015. The soil at the site consists of 20% clay, 43% silt, 33% sand, and 4% organic material. The field is 8 m above sea level and does not slope.

### Experimental Plan

The same experimental design was used in both years: a strip-plot design (Gomez and Gomez, 1984) with four replicates. Four factors were investigated in factorial complete blocks for a total of 32 treatment combinations. These four factors were: (1) companion crop (no companion crop, grass, clover or a grass/clover mixture), (2) repeated mowing, (3) early rhizome fragmentation (ERF: before companion crop sowing), and (4) late rhizome fragmentation (LRF: a few weeks after crop sowing). The companion crop and ERF treatments were randomized in columns, and mowing and LRF were randomized in rows. Total plot size was 4 m × 4 m in 2014 and 3 m × 3 m in 2015.

White clover (*Trifolium repens* L. cv. Klondike) and Italian ryegrass (*Lolium multiflorum* Lam. cv. Fredrik) were sown at 12–13 cm row spacing and at a seed rate of 10 and 20 kg ha^-1^, respectively, in the pure stands and 5 and 10 kg ha^-1^, respectively, in the mixed stands. No fertilizer was added.

Mowing to 3–5 cm height was performed across the entire plot, using a regular rotary lawnmower, when the average *E. repens* shoot had approximately 3–4 leaves. This resulted in 10 mowings in 2014 and eight mowings in 2015, with an average mowing interval of 16 and 13 days, respectively (**Table 1**). The cut material was left in the field. Soil water concentration was measured using a soil moisture sensor (ThetaProbe type ML2x, Delta-T Devices, England) 10 times in each block at each mowing (Appendix A).

**Table 1:** Dates of early (ERF) and late rhizome fragmentation (LRF), crop sowing, and mowing in 2014 and 2015.

	2014	2015
ERF	May 14	May 16
Crop sowing	May 16	May 20
LRF	July 7	July 9
Mowing	June 5	June 17
	June 19	June 29
	June 25	July 6
	July 7	July 20
	July 21	August 3
	August 8	August 17
	August 19	August 31
	September 1	September 21
	September 15	
	October 6	


*ERF was performed pre-sowing, LRF in the growing crop.*

Rhizome fragmentation was achieved using a Kverneland prototype (Kverneland Group Operations, Norway) that is fitted with vertical coulter disks taken from a plow. The disks were run through the soil, cutting rhizomes and roots, but causing minimal disturbance to the soil (see Appendix B). The vertical disks were set 10 cm apart and the machine was 1.5 m wide. The machine was run through the center of the plots and then again perpendicularly to the first run, resulting in a grid pattern. In 2014, ERF reached 10 cm depth, but LRF only reached an estimated 8 cm depth, due to hard soil conditions. In 2015, ERF reached 12–14 cm, while LRF reached 8–9 cm. Treatment dates are given in **Table 1**.

A few shoots of *Cirsium arvense* emerged in some of the plots and were cut at soil level with scissors in August, to avoid confounding effects due to thistle interference.

### Sampling

Three methods were used to measure *E. repens* abundance: shoot number, rhizome biomass, and shoot biomass. In addition to *E. repens* abundance, the nitrogen (N) concentration in the *E. repens* shoots was measured and the companion crop biomass was harvested. Sampling focused on the treatment combinations that tested the initial hypotheses (**Table 2**).

**Table 2:** Sampling/counting procedures conducted in the treatment combinations with early (ERF) and late rhizome fragmentation (LRF), their designation number, and the corresponding dates.

		No fragment	ERF	LRF	ERF+LRF
Mown	No crop	2–3, 5–7	2–3	2–3	2–3
	White clover (WC)	2–3, 5–7	2–3	2–3	2–3
	Italian ryegrass (IR)	2, 5–7	2		
	WC+IR	2, 4, 5–7	2, 4, 6–7	4, 6–7	4, 6–7
Not mown	No crop	1–2	1–2	2	2
	WC	1–2	1–2	2	2
	IR				
	WC+IR	4, 6–7	4, 6–7	4, 6–7	4, 6–7

	**Sampling/counting**		**Designation**	**2014**	**2015**

	Shoot numbers, occasion 1		1	June 5	June 16
	Shoot numbers, occasion 2		2	July 2	July 1
	Shoot numbers, occasion 3		3	August 4	August 3
	Early rhizome biomass		4	August 5	August 6
	Shoot N content		5	September 22	August 7
	Shoot biomass		6	October 27	November 9
	Late rhizome biomass		7	October 27	November 23


The number of *E. repens* shoots (including both main shoots and tillers) with at least one fully developed leaf was counted in the center 0.8 m × 0.8 m (0.64 m^2^) of the plot on three different occasions: (1) just before the first mowing, (2) prior to the third mowing and LRF, and (3) in August, prior to the first rhizome sampling.

Rhizome biomass was sampled in the beginning of August and in October–November. In August, a 0.8 m × 0.8 m (0.64 m^2^) frame was placed in the center of all grass–clover mixture treatment plots. A total of eight samples were taken around the outside of the frame, two on each side, using a golf hole drill (10.5 cm diameter, 21 cm depth). All rhizomes from the samples were collected and cleaned with water, and fresh weight was recorded. A sample of the rhizomes was randomly selected for a germination test (see below), and the rest were dried for 24 h at 105°C and the dry weight was recorded. The dry weight of the rhizomes taken for the germination test was estimated based on the difference between fresh weight and dry weight of the other rhizomes. In October–November, the center 0.8 m × 0.8 m (0.64 m^2^) of plots was dug up in all the grass–clover mixture treatments and in the companion crop plots that were mown, but where rhizome fragmentation was not performed (**Table 2**). The rhizomes were dried at 50°C for 48 h and dry weight was recorded.

In October–November, prior to the late rhizome sampling, all aboveground biomass was cut at soil level with scissors in the center 0.8 m × 0.8 m (0.64 m^2^) in all grass–clover mixture treatments and in the other companion crop plots that were mown, but where rhizome fragmentation was not performed (**Table 2**). The aboveground biomass was divided into Italian ryegrass, white clover, *E. repens*, and other weeds, and then dried at 50°C for 48 h.

In autumn (**Table 2**), *E. repens* shoots were randomly collected from the center 0.8 m × 0.8 m (0.64 m^2^) of all mown plots without rhizome fragmentation. The shoots were dried at 50°C for 48 h and analyzed for N content by combustion on an elemental analyzer (Leco CNS-2000, Leco, Corp., St. Joseph, MI, United States).

### Germination Test

The rhizome material sampled in early August was cut into 5-cm pieces with at least one node. Twenty rhizome pieces were randomly selected from each plot, excluding old and young rhizome fragments. Their combined fresh weight was recorded.

Two filter papers were placed in the bottom of Petri dishes (90 mm) and sprayed with 5 mL of distilled water. Four rhizome pieces were placed in each Petri dish, with a total of five dishes per plot. The Petri dishes were then sealed with paraffin film, covered in aluminum foil and placed in a climate chamber for 2 weeks. The temperature in the climate chamber was 17°C during day (16 h) and 9°C at night (8 h). After 2 weeks, *E. repens* shoot and rhizome biomass were separated and then dried at 105°C for 24 h.

### Statistical Analysis

The results were analyzed in mixed linear models with main effects (companion crop, ERF, LRF, and mowing) and their interactions as fixed factors and year, block, rows, and columns as random factors. As not all treatment combinations were sampled for each measure, the main effects had to be analyzed in separate models (**Tables 3**, **4**). Rhizome biomass was analyzed as a repeated measure, with variance components as the covariance structure. Tukey–Kramer tests and contrasts were used for post-analysis. All analysis was performed using proc GLIMMIX in SAS 9.4 (SAS Institute, Inc.).

**Table 3:** Analysis of variance (ANOVA) table of *E. repens* shoot numbers on occasion 2 and 3, rhizome dry weight (DW) and shoot DW in the field experiment, and initial rhizome fresh weight (FW) and shoot DW in the germination test.

	Shoot numbers,	Shoot number,	Rhizome	Shoot	Initial rhizome FW	Shoot DW
	occasion 2	occasion 3	DW	DW	(Germination test)	(Germination test)
ERF	**0.01**	**0.0002**	**0.02**	0.5	** 0.009**	0.1
LRF		0.5	**0.001**	**0.02**	** <0.0001**	0.7
ERF^∗^LRF		0.8	0.5	0.5	0.9	0.8
Mow	** 0.002**		**<0.0001**	** <0.0001**	** <0.0001**	1
ERF^∗^Mow	1		** 0.0002**	0.6	0.2	0.4
LRF^∗^Mow			**0.003**	**0.02**	** 0.02**	0.3
ERF^∗^LRF^∗^Mow			0.1	0.6	0.6	0.4
Sampling time			**<0.0001**			
ERF^∗^Time			**0.04**			
LRF^∗^Time			0.7			
ERF^∗^LRF^∗^Time			0.1			
Mow^∗^Time			**<0.0001**			
ERF^∗^Mow^∗^Time			0.1			
LRF^∗^Mow^∗^Time			0.4			
ERF^∗^LRF^∗^Mow^∗^Time			1			
Initial rhizome FW						**0.0002**


*The fixed variables early rhizome fragmentation (ERF), late rhizome fragmentation (LRF), mowing, and their interactions were investigated, depending on whether they were sampled in all treatments (**Table 2**). Rhizome DW was analyzed as a repeated measure since it was sampled twice in the same plots during autumn. Random variables are not shown. Missing values indicate that the term was not included in the analysis. *P*-values ≤ 0.05 are in bold.*

**Table 4:** Analysis of variance table of the statistical model used to analyze *E. repens* shoot numbers on occasion 1–3, late rhizome dry weight (DW), shoot DW and shoot nitrogen (N) concentration from the field experiment.

	Shoot number,	Shoot number,	Shoot number,	Late	Shoot	N%
	occasion 1^∗^	occasion 2^∗^	occasion 3^∗^	rhizomes	DW	
Crop	0.5	**<0.0001**	0.4	**0.04**	**0.007**	**0.001**
ERF	**0.03**	**0.009**	**0.0002**			
Crop^∗^ERF	0.2	0.7	0.9			


*The fixed variables early rhizome fragmentation (ERF) and crop were analyzed. Random variables are not shown. Missing values indicate that the term was not included in the analysis. *P*-values ≤ 0.05 are in bold.*

*^∗^All crops compared at Occasion 2, but only white clover and no companion crop compared at Occasions 1 and 3.*

## Results

### Plant Growth

In October–November, *E. repens* rhizome (628 vs. 330 g m^-2^; *P* < 0.001) and shoot biomass (236 vs. 144 g m^-2^; *P* = 0.01) was almost twice as great in 2014 as in 2015, while total shoot biomass (crops+*E. repens*+other weeds) was similar (363 vs. 318 g m^-2^; *P* = 0.1) in the grass–clover control plots (no mowing or rhizome fragmentation). In the grass–clover mixture control plots, the white clover shoot biomass was higher in 2014 than in 2015 (14.4 g vs. 1.2 g m^-2^; *P* = 0.01), while the opposite was the case for Italian ryegrass (29 vs. 89 g per g m^-2^; *P* < 0.001). The amount of white clover and Italian ryegrass in pure stands was only measured in the mown plots without rhizome fragmentation (**Table 2**). In the pure stands, there was more white clover biomass than in the grass–clover mixture (63 vs. 33 g m^-2^; *P* = 0.06), but no difference in Italian ryegrass biomass (87 vs. 90 g m^-2^; *P* = 0.9).

### Rhizome Fragmentation Effect

**FIGURE 1:**
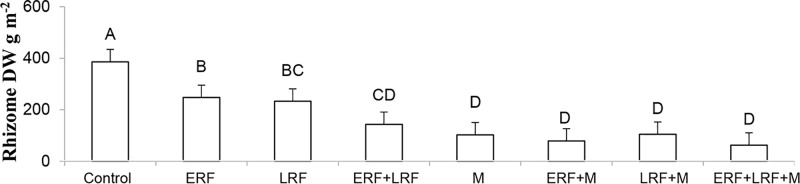
Results for the Italian ryegrass-white clover mixture. The graphs show the effect of early (ERF) and late rhizome fragmentation (LRF) and mowing (M) on *Elymus repens* rhizome dry weight (DW) in autumn. The results are the average over both years and both sampling times. Error bars show standard error. Letters show the result of Tukey–Kramer grouping at α = 0.05.

**FIGURE 2:**
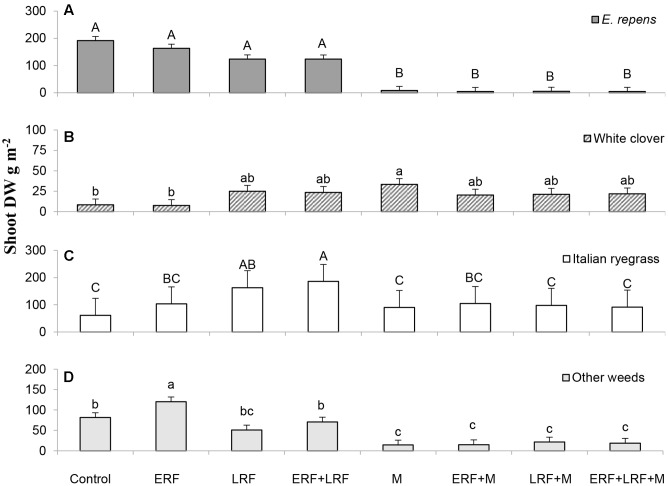
Results for the Italian ryegrass-white clover mixture. The graphs show the effect of ERF and LRF and mowing (M) on shoot dry weight (DW) of: **(A)** *E. repens*, **(B)** white clover, **(C)** Italian ryegrass, and **(D)** weeds other than *E. repens*. Error bars show standard error. Letters show the result of Tukey–Kramer grouping at α = 0.05. Note the difference in scale on the y-axis.

**FIGURE 3:**
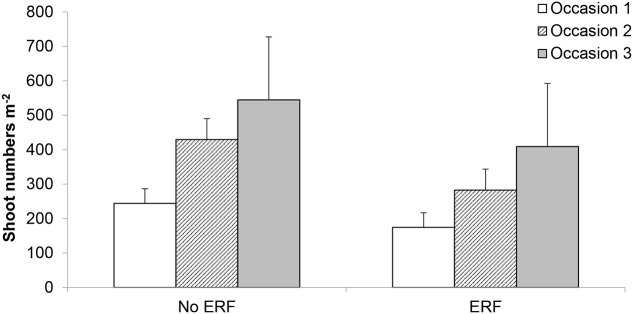
Results for the white clover and no crop plots. Effect of ERF, which significantly reduced *E. repens* shoot numbers compared with no-ERF, on three sampling occasions during summer (occasions 1–3; **Table 2**). Shoot sampling 1 was conducted in the non-mown plots, sampling 3 in the mown plots, and sampling 2 in both (**Table 2**). Error bars show standard error.

Using vertical coulter disks to fragment *E. repens* rhizomes had a detrimental effect on *E. repens* rhizome (**Figure 1**) and shoot biomass acquisition (**Figure 2**), and number of shoots (**Figure 3**), but it benefited the growth of the sown crops, especially Italian ryegrass (**Figures 2B,C**). On all three sampling occasions during summer, there were about 30% fewer *E. repens* shoots in ERF plots than in plots without ERF (**Figure 3**). In autumn, *E. repens* rhizome biomass was, on average, 38% lower in the ERF and LRF treatments than in the control (**Figure 1**). Two rhizome fragmentations (ERF+LRF) resulted in less rhizome biomass than a single fragmentation (contrast between ERF and LRF, and ERF+LRF; *P* < 0.0001; **Figure 1**), on average two rhizome fragmentations resulted in 63% less rhizome biomass than in the control (**Figure 1**). Moreover, rhizome fragmentation by vertical coulter disks significantly reduced *E. repens* shoot biomass compared to control (contrast between ERF, LRF and ERF+LRF, and control; *P* < 0.0001; **Figure 2A**).

Using the vertical coulter disks in the grass–clover mixture increased the total shoot biomass (crops+*E. repens*+other weeds) by 30% with LRF (*P* = 0.06) and 50% with ERF+LRF (*P* = 0.04; data not shown). In the grass–clover mixture, LRF and ERF increased the Italian ryegrass shoot biomass compared to control (78% by ERF, 170% by LRF and 200% by ERF+LRF; **Figure 2C**). LRF and ERF+LRF increased white clover shoot biomass compared to control (contrast, *P* = 0.04). ERF increased the shoot biomass of weeds other than *E. repens* by 48% (*P* = 0.009; **Figure 2D**).

### Effect of Mowing with or without Rhizome Fragmentation

Repeated mowing reduced *E. repens* rhizome biomass by about 75% compared with the grass–clover control (**Figure 1**). Combining mowing with rhizome fragmentation did not significantly lower rhizome biomass compared to mowing alone (**Figure 1**).

In the grass–clover mixture, repeated mowing reduced the total shoot biomass (crops+*E. repens*+other weeds) by 35% compared to control and *E. repens* shoot biomass by 95%, while significantly increasing the white clover biomass from 8 to 33 g m^-2^ (**Figure 2B**). Combining mowing with LRF did not produce as much Italian ryegrass as LRF without mowing (*P* < 0.001; **Figure 2C**), or as much white clover as mowing without LRF (*P* = 0.03; **Figure 2B**).

### Companion Crop Effect

The effect of companion crop species on *E. repens* was only investigated in the mowing treatments (**Table 2**). There, pure white clover tended to reduce *E. repens* shoot biomass (*P* = 0.08) and rhizome biomass (*P* = 0.06) compared with the control (**Tables 4**, **5**). Pure Italian ryegrass reduced *E. repens* shoot biomass by 85% and shoot numbers by 53% compared with the control, but had no significant effect on rhizome biomass (**Tables 4**, **5**). The *E. repens* shoots that grew in the pure Italian ryegrass stands had about 0.4–0.5% points lower N concentration than those that grew in the pure white clover stands or in the control. The grass–clover mixture reduced *E. repens* shoot biomass, shoot numbers, and shoot N content to a similar degree as pure Italian ryegrass, but also reduced *E. repens* rhizome biomass, similar to the effect of white clover (**Table 5**). The land equivalent ratio (calculated as in Saia et al., 2016) in the grass–clover mixture was 1.57 compared to the pure stands, which shows that the mixture produced more biomass than the pure stands.

**Table 5:** Effect of companion crops on *E. repens* shoot numbers on occasion 1–3, shoot nitrogen (N) content, rhizome, and shoot dry weight (DW), and shoot DW of weeds other than *E. repens* (see **Table 2** for sampling dates).

	No crop	White clover	Italian ryegrass	Mixture
*E. repens* shoot number, occasion 1	217 (42) a	202 (42) a	–	–
*E. repens* shoot number, occasion 2	322 (54) a	344 (54) a	198 (54) b	209 (54) b
*E. repens* shoot number, occasion 3	492 (183) a	461 (183) a	–	–
*E. repens* shoot N content	4.7 (0.14) ab	4.8 (0.14) a	4.3 (0.14) c	4.5 (0.14) bc
*E. repens* rhizome DW	192 (23) a	129 (23) ab	152 (23) ab	126 (23) b
*E. repens* shoot DW	31 (7) a	13 (7) ab	5 (7) b	8 (7) b
Other weed shoot DW	65 (11) a	45 (11) a	12 (11) b	14 (11) b


*Mean shoot numbers and DW are given as m^-*2*^ and shoot N content as % g DW^-*1*^, with standard error in brackets. Letters show the result of a Tukey–Kramer test at α = 0.05.*

### Germination Test

Both rhizome fragmentation and mowing reduced the amount of shoot biomass produced by the rhizomes in the germination test by 20–30%. However, the initial rhizome fresh weight was also 20–30% lower in the rhizomes from the treatments than in those from the control, and initial rhizome fresh weight was correlated with the shoot biomass produced by the rhizomes in the germination test (*P* < 0.001, slope est. 0.02; **Table 3**). When initial rhizome fresh weight was accounted for, mowing, ERF and LRF no longer had a direct effect on the shoot weight produced.

## Discussion

The results of this study support the hypothesis that fragmenting *E. repens* rhizomes reduces its growth and increases the growth of a grass–clover companion crop. The rhizome fragmentation was sufficient to weaken *E. repens* and shift the competitive balance toward the crop, which also supports the suggestion that perennial plants derive significant advantage from their vegetative networks in mixed plant stands (Stuefer et al., 2004; Song et al., 2013; Lopp and Sammul, 2016). Some of the reduction in rhizome biomass and shoot numbers can be explained by direct rhizome losses, i.e., some fragments become too small to produce shoots that can reach the surface or be competitive enough to survive (Håkansson, 1968). However, the germination test showed that the effect of rhizome fragmentation went beyond that. There was not only a reduction in total rhizome biomass, but also in weight per cm rhizome. Thus, the rhizome fragmentation resulted in lower *E. repens* competitiveness, which in turn resulted in less energy being stored in the rhizomes. As rhizome weight per cm rhizome was strongly correlated with how much shoot biomass the rhizomes produce, this means that rhizome fragmentation reduced both the rhizome biomass production, and the overall fitness of the rhizome network. As the storage organs of perennial plants lose a great deal of weight during winter due to respiration (Verwijst et al., 2013), the lower weight per cm rhizome may also result in higher winter mortality.

Of the great number of factors that could have reduced the competitive ability of *E. repens*, e.g., slower expansion, increased intraspecific competition, increased susceptibility to infectious diseases, the most obvious of these would be changes in the expansion capabilities of *E. repens*. While the biomass production of perennial weeds per m^2^ does not necessarily diminish due to fragmentation of their underground organs under conditions of no or little competition from other species, it can drastically influence the capacity of each individual plant to acquire resources and propagate. For example, Anbari et al. (2016) found that plants from longer root fragments of *Sonchus arvensis* produced more flower heads than those from shorter fragments, even though the total number of flower heads per m^2^ was the same.

Rather than damaging the crop, the use of the vertical disks increased crop shoot biomass. The reduced competitiveness of *E. repens* must undoubtedly have played a significant role in increasing the crop biomass. However, as there was a particularly high increase in crop biomass when the vertical disks were used in the growing crop (without having a greater reductive effect on *E. repens* than when performed pre-sowing), the results indicate that there are also some positive direct effects of the vertical disk treatment on the crop. For example, the disks could have opened up the stand and enabled greater light penetration. In addition, although the soil disturbance caused by the vertical disks was minimal (Appendix B), it may have increased the available oxygen and N mineralization. On golf courses, it is common practice to aerate the soil in order to increase the decomposition rate of organic matter, and consequently improve grass growth (Smith, 1979; Huang et al., 1998). Alternatively, the fact that LRF plots had more crop biomass than ERF plots could be explained by the fact that there was more weeds other than *E. repens* in the ERF crops. Most likely, this was because more annual weeds germinated in response to the soil disturbance caused by the rhizome fragmentation, when it was performed in spring (Håkansson, 2003). In summer, when LRF was performed, germination of annual weeds would most likely be lower and the weeds that emerged would have to compete with an established crop. Distinguishing the effects of weed competition from the direct effects of the vertical disk treatments requires comparisons with plots without any weed competition, and such control plots were not included in the present study.

There was no support for the hypothesis that repeated mowing can improve the efficacy of rhizome fragmentation. Independently, both rhizome fragmentation and mowing had a negative influence on *E. repens*. However, the difference between fragmentation combined with mowing, and mowing alone was not significantly different (similar to Bergkvist et al., 2017). Potentially, the high mowing frequency used in the study, which strongly suppressed *E. repens*, may have hidden potential interactions. If so, rhizome fragmentation combined with a more realistic mowing regime (2–4 times per year) may provide adequate control of *E. repens*. The difference in selectivity between mowing and rhizome fragmentation was reflected in the effect these treatments had on the crops. Mowing decreased the total biomass of both grass species, even if proportionally *E. repens* lost more shoot biomass than Italian ryegrass (**Figure 2**). On the other hand, mowing increased white clover shoot biomass. This is likely the result of the horizontal growth pattern of white clover, which enables it to survive and benefit from mowing better than most grasses, and also to benefit from the reduced light competition from the grasses (Burdon, 1983). In contrast to mowing, rhizome fragmentation reduced *E. repens* biomass production, while increasing total crop biomass. Of course, not all crops may respond as favorably at all times. For example, the stolons of white clover would likely have been damaged by the vertical disks, but in the current experiment white clover had not produced stolons by the time of the treatments.

There was some support for the hypothesis that clover competition reduces *E. repens* rhizome biomass more, and shoot biomass less, in relative terms than competition from Italian ryegrass. This strengthens the theory that, at least in the short-term, clovers are more efficient than grasses at reducing *E. repens* rhizome biomass, while grasses have a larger relative effect on *E. repens* shoot biomass (Ringselle et al., 2015, 2017). The study also support previous findings that a mixture of clover and grass is superior to pure stands as the mixture produces more biomass per land area and combines the competitive advantages of each species, while mitigating their weaknesses (Ringselle et al., 2015). We did not investigate the interaction between resource competition type and rhizome fragmentation, but Håkansson (1971) found that white mustard, which competes strongly for N, suppresses plants from heavily fragmented rhizomes more than plants from less fragmented rhizomes. In comparison, Kolberg et al. (2017) did not find that N_2_-fixing white clover competed better against plants from more fragmented rhizomes than less fragmented rhizomes.

### Implications for Perennial Weed Control

The idea of controlling perennial weeds by fragmenting their clonal networks is not new, but so far it has been achieved through non-selective, intense tillage. One of the advantages of the method used in this experiment is that it enables study of rhizome fragmentation under field conditions in relative isolation from other effects of tillage (e.g., rhizome displacement and shoot destruction). The present experiment showed that using vertical disks makes it possible to selectively target the belowground storage organs of perennial weeds, such as *E. repens*. There are several aspects of agronomic interest. First, there was no loss in weed control efficacy when selective fragmentation was performed in the growing crop. In fact, it was more beneficial to the crop than when done pre-sowing. The ability to use the method in a growing crop gives farmers far more flexibility than if it has to be conducted pre-sowing or post-harvest. Moreover, it makes it possible to combine the method with other operations (e.g., fertilization), and thus increase the overall resource efficiency of the system. Second, the effect was cumulative when used multiple times during the season. Thus, both timing and number of treatments can be optimized to achieve the best control efficiency. Third, the method is seemingly not reliant on mowing, but can be successfully combined with mowing. This drastically increases the flexibility of the method, as it does not rely on another treatment to be successful and optimization of the method can be performed without necessarily taking another factor into account. It also means that it can potentially be used in an annual crop where mowing would be detrimental to crop yields. Fourth, the effect was relatively fast, immediately reducing *E. repens* shoot numbers and reducing rhizome biomass per cm rhizome already in early autumn (**Figure 1**). Thus, it may be possible to utilize the method in the year that the grass–clover crop is plowed under and it could therefore be followed by an autumn-sown annual crop.

The method has great potential, but there are also many unanswered questions. The present experiments were conducted on a soil with a reasonably low clay content (20%). It is possible that soil type could affect the efficiency of the method, e.g., by changing how easily the disks can penetrate the soil or by influencing the effect the treatment has on the crop. For instance, aerating the soil may have an even greater positive effect on the crop on heavy clay soils (Bhattarai et al., 2006). Other perennial species may also react differently than *E. repens*. For example, stolon fragments would not have to use as much energy to reshoot as rhizomes that are buried in the soil but, on the other hand, they are less protected from the elements. The control efficacy may also be influenced by the age of the ley or grassland. It has been suggested that rhizomes grow closer to the soil surface in older leys (Cussans, 1973), which means that more rhizomes would be fragmented by the disks, but also that they would need less energy to reach the surface. In an older ley, the *E. repens* rhizomes would not be weakened by recent plowing and harrowing, as was performed prior to the present experiment. The higher N availability in older mixed leys (Clement and Williams, 1967), may also reduce the efficacy of the method as a high N availability appears to benefit smaller rhizome fragments more than larger fragments (Turner, 1966).

## Conclusion

• Rhizome fragmentation through vertical disks has a reductive effect on *E. repens* rhizome and shoot biomass acquisition and increases the shoot biomass of grass–clover companion crops.
• The detrimental effect of rhizome fragmentation on *E. repens* is unaffected by whether it is performed pre-sowing or in the growing crop, and it is cumulative with number of applications. Fragmentation in the growing crop results in more companion crop biomass than when performed pre-sowing.
• Repeated mowing performed during the growth season has a strong reductive effect on both *E. repens* and the companion crops.
• White clover tends to have a relatively larger effect on rhizomes compared with shoots than Italian ryegrass, while a white clover-Italian ryegrass mixture seems to combine the effect of the component crops.

## Author Contributions

GB, LB, EM, and KM were responsible for the conceptualization of the treatments and the design of the experiments. BR and EB performed the data synthesis and analysis. EM and EB performed the field and lab work. EB wrote a first draft based on initial data, which BR brought to completion with significant input and criticism from the other authors.

## Conflict of Interest Statement

The authors declare that the research was conducted in the absence of any commercial or financial relationships that could be construed as a potential conflict of interest.
