# Supplementary material for: Rhizome Fragmentation by Vertical Disks Reduces Elymus repens Growth and Benefits Italian Ryegrass-White Clover Crops
Source: Front Plant Sci. 2018 Jan 11;8:2243. doi: 10.3389/fpls.2017.02243 (PMC5769382; doi:10.3389/fpls.2017.02243)
Supplement: Supplementary file 1 [file Data_Sheet_1.DOCX]

**Appendix A** Average soil water content (10 samples per block), measured during the experiment in conjunction with mowings, in both the mown and the unmown plots (5 samples in each). For exact mowing and sampling dates, see Tables 1 and 2.
