# Supplementary material for: Rhizome Fragmentation by Vertical Disks Reduces Elymus repens Growth and Benefits Italian Ryegrass-White Clover Crops
Source: Front Plant Sci. 2018 Jan 11;8:2243. doi: 10.3389/fpls.2017.02243 (PMC5769382; doi:10.3389/fpls.2017.02243)
Supplement: Supplementary file 2 [file Data_Sheet_2.DOCX]

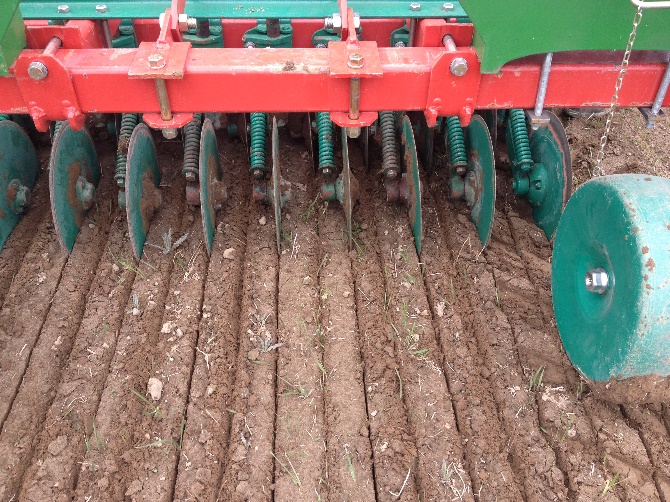

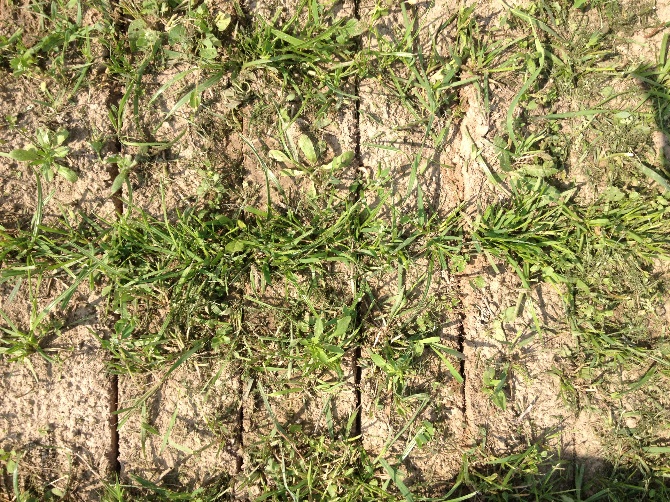


A B

**Appendix B** Kverneland prototype fragmenting rhizomes at: A) pre-sowing and B) in the growing crop.
